# Eisenmenger Syndrome in Pregnancy: A Challenging Case With Important Implications for Primary Care and Maternal Mortality Reduction

**DOI:** 10.7759/cureus.115314

**Published:** 2026-08-27

**Authors:** Anupma Kumari, Indira Prasad, Neeraj Kumar, Shyama Shyama, Asiya A Ismail

**Affiliations:** 1 Obstetrics and Gynaecology, All India Institute of Medical Sciences, Patna, Patna, IND; 2 Anaesthesiology, All India Institute of Medical Sciences, Patna, Patna, IND; 3 General Medicine, All India Institute of Medical Sciences, Patna, Patna, IND

**Keywords:** case report, eisenmenger`s syndrome (es), pregnancy, pulmonary arterial hypertension (pah), ventricular septal defect (vsd)

## Abstract

Eisenmenger syndrome (ES) represents the advanced form of pulmonary arterial hypertension (PAH) associated with a reversed or bidirectional shunt at the atrioventricular or aortopulmonary level. Pregnant women have a higher risk of developing volume overload due to this syndrome, compounded by the physiological changes of pregnancy, which can result in rapidly progressive cardiopulmonary decompensation and thrombotic complications. Due to the substantial risk to both the mother and fetus, women are advised not to conceive or to terminate the pregnancy in the first trimester. However, with appropriate management and careful consideration of all potential risks, positive neonatal and maternal outcomes can be achieved. We report a case of an 18-year-old primigravida with PAH and preeclampsia at 35+5 weeks' period of gestation (POG), who presented to our center for the first time with lower limb and vulval swelling.

## Introduction

A very rare condition in pregnancy, Eisenmenger syndrome (ES) was first described in 1897 by Victor Eisenmenger [1]. As one of the absolute contraindications to pregnancy, ES develops from a long-standing left-to-right shunt associated with congenital heart defects, including ventricular septal defect (VSD), atrial septal defect (ASD), and patent ductus arteriosus (PDA), which eventually leads to the development of pulmonary arterial hypertension (PAH). This syndrome may also occur in unrepaired tetralogy of Fallot (TOF). Without early surgical intervention, reversal of a left-to-right shunt may result in a bidirectional or right-to-left shunt [2]. ES carries a high maternal mortality rate ranging from 30% to 50%, which may increase to as high as 65% following cesarean section [3]. This syndrome is associated with a heightened risk of progressive cardiopulmonary failure, thrombotic complications, and sudden cardiac death. Consequently, women are typically counseled against pregnancy, and termination of pregnancy during the early stages is often recommended [4]. Management of ES during pregnancy is challenging and involves a multidisciplinary approach [5]. In this report, we present a case of ES secondary to VSD with PAH in an 18-year-old primigravida who underwent a cesarean section at 35+5 weeks of gestation and delivered a live baby girl weighing 1.560 kg with an Apgar (Appearance, Pulse, Grimace, Activity, and Respiration) score of 8/10.

## Case presentation

**Figure 1:**
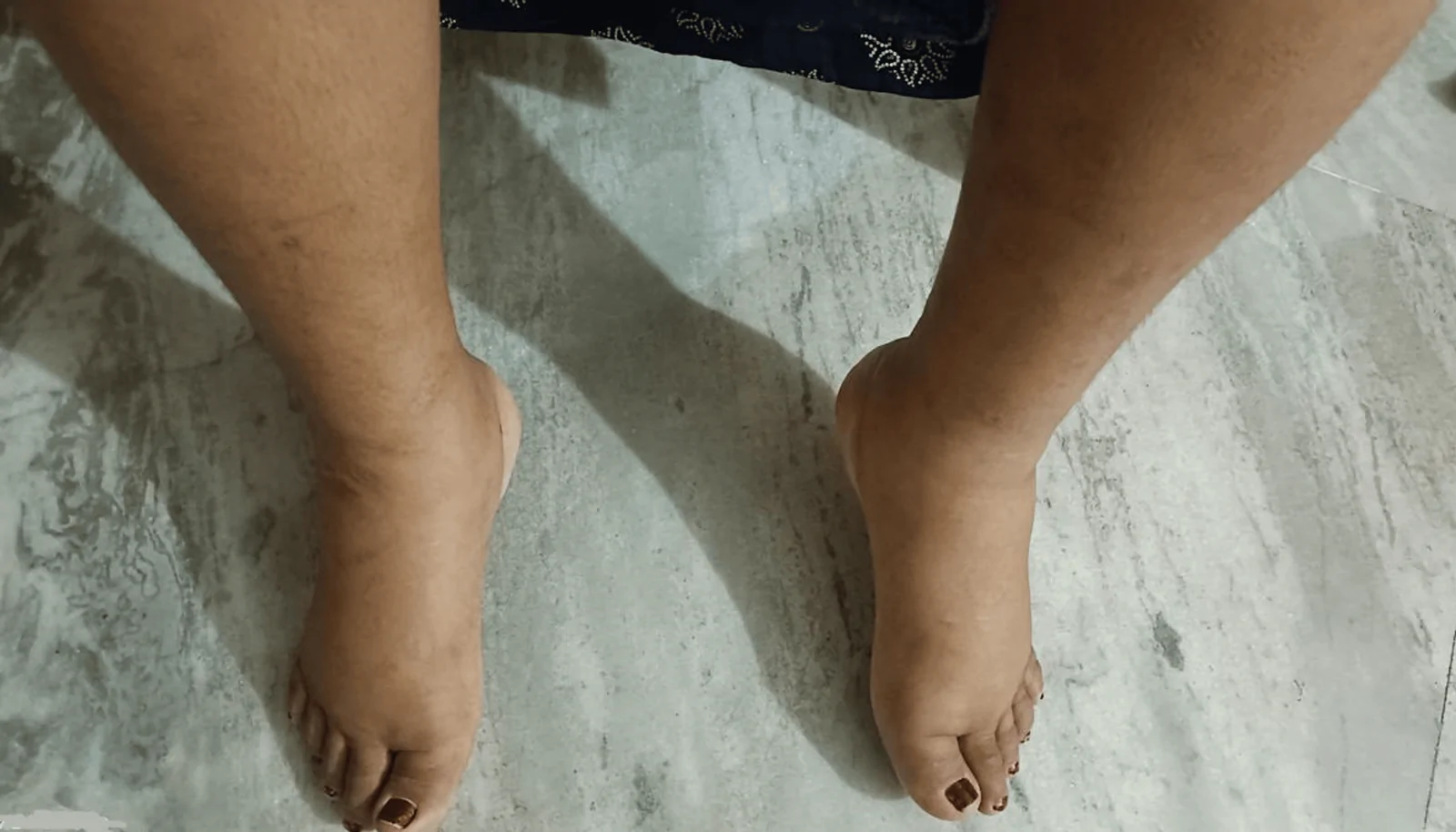
Gross edema in both lower limbs

An 18-year-old primigravida presented to our gynaecology emergency unit at 35 + 5 weeks' period of gestation (POG) with complaints of generalized swelling of the body and breathlessness. She had experienced dyspnea on exertion in childhood; however, no medical treatment had been sought, and her cardiac condition had remained undiagnosed until she consulted a local practitioner two weeks previously. Echocardiography (ECHO) at that time showed VSD with PAH, following which she was referred to our tertiary-level institution. On physical examination, pallor and central cyanosis were noted, and gross edema of the bilateral lower limbs (Figure 1) and vulva was present.

**Figure 2:**
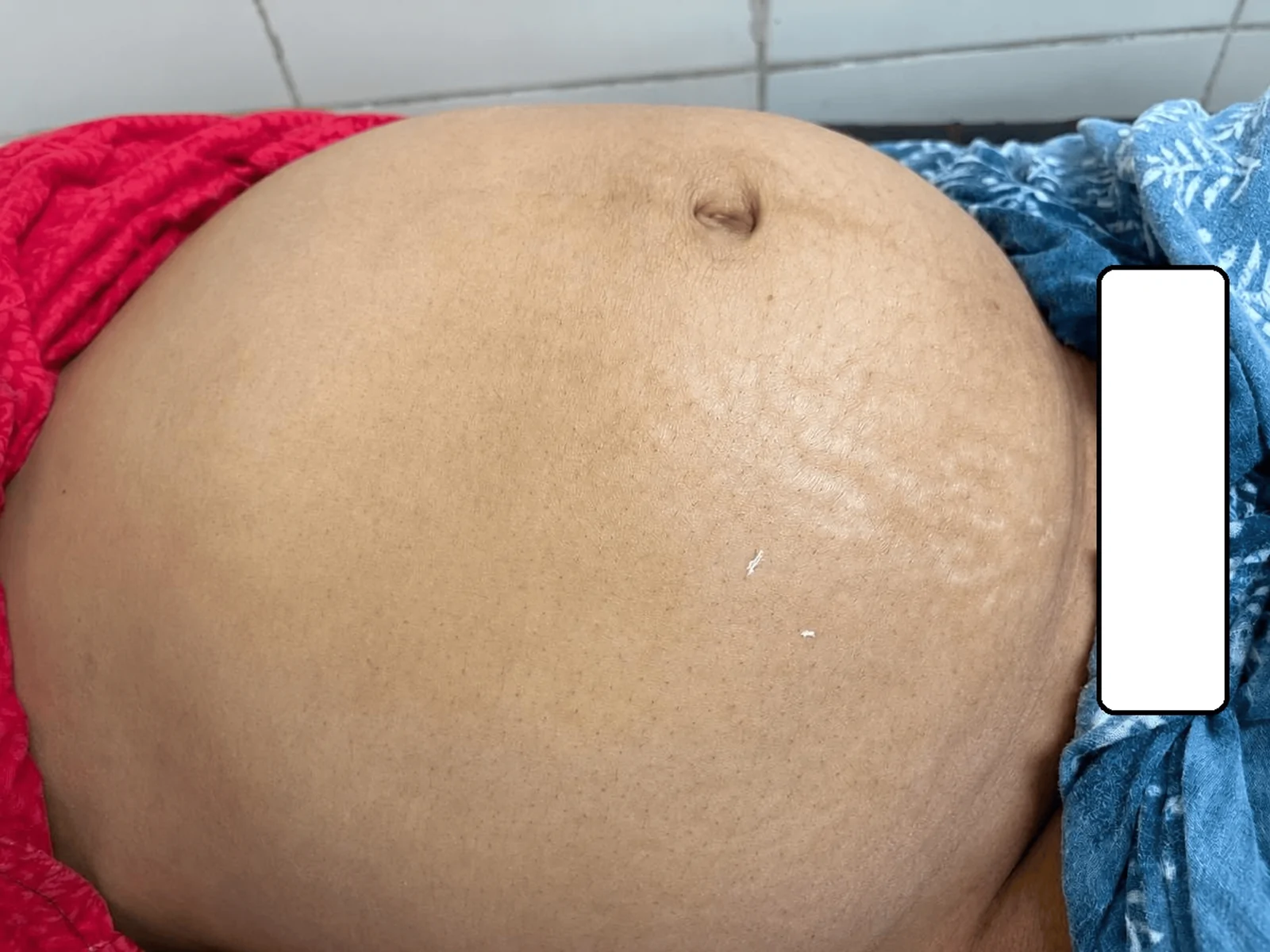
Gravid uterus with abdominal wall edema

**Figure 3:**
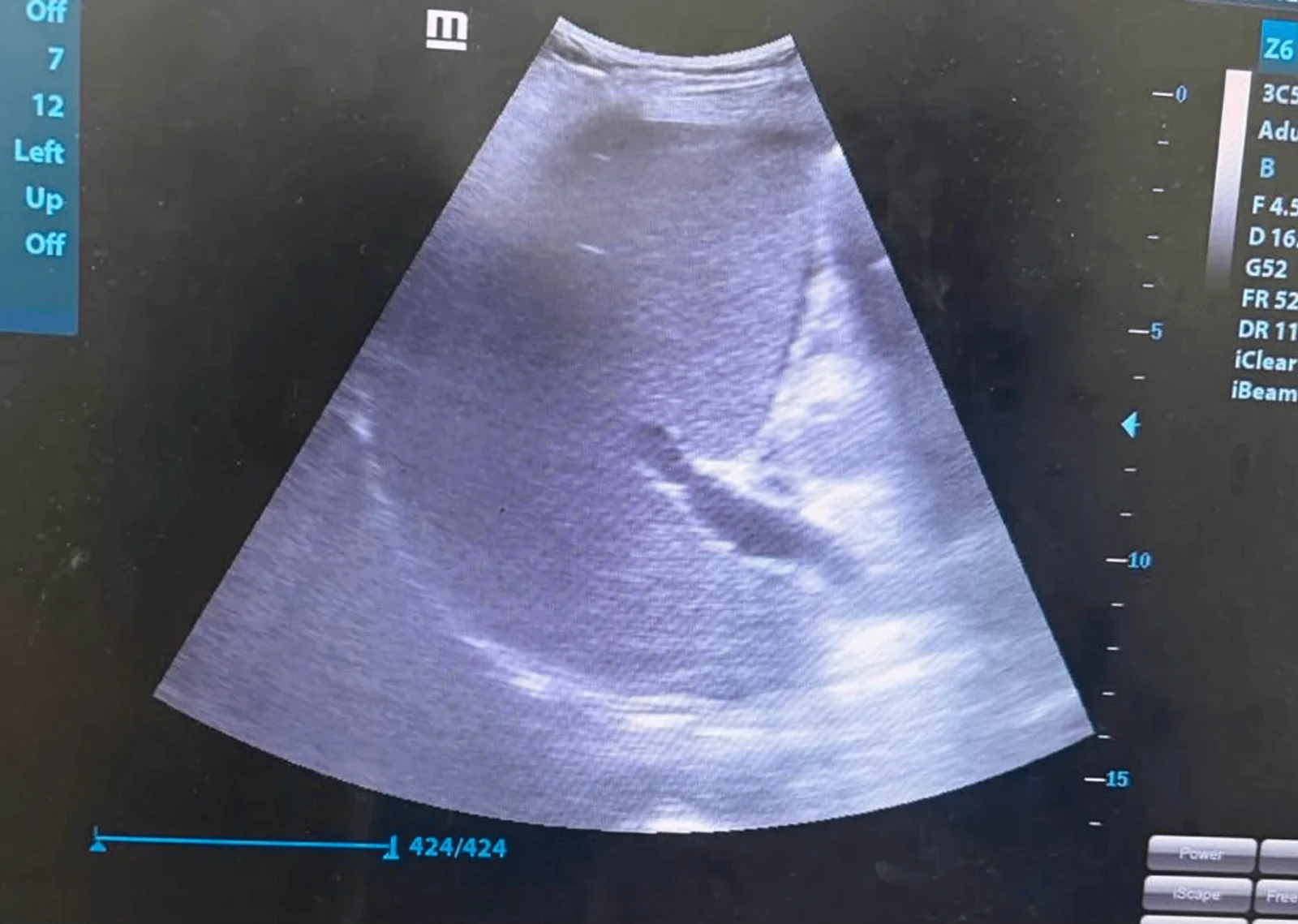
Abdominal ultrasound showing free fluid in the subhepatic region

**Figure 4:**
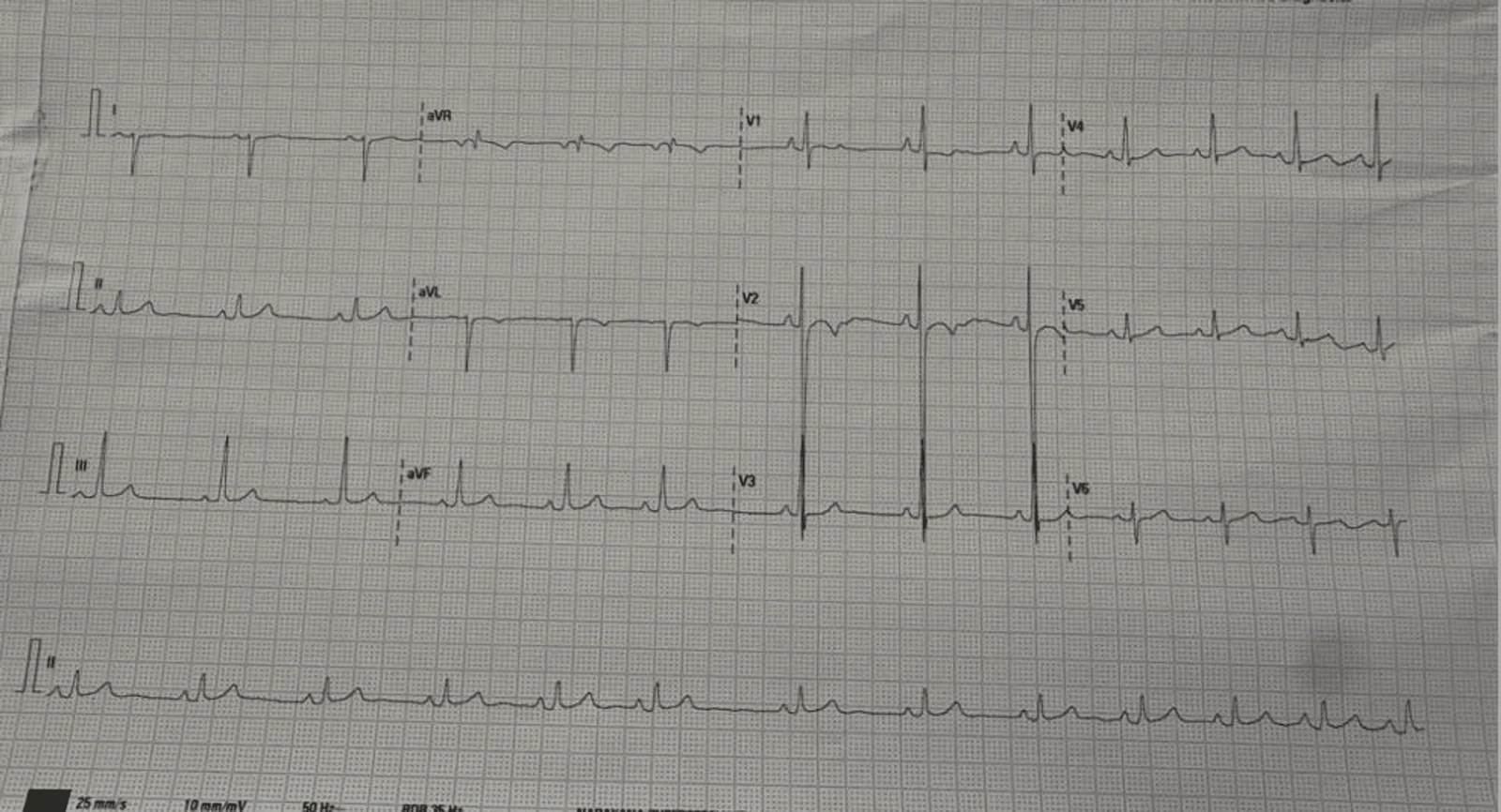
ECG showing RAD and RVH ECG: electrocardiogram; RAD: right axis deviation; RVH: right ventricular hypertrophy

The patient was afebrile, with a pulse rate of 88 beats/min and a blood pressure of 150/110 mmHg. On room air, pulse oximetry showed an oxygen saturation of 92%; therefore, oxygen was administered by mask at 5 L/min, with regular monitoring of oxygen saturation. Chest auscultation revealed bilateral vesicular breath sounds with crepitations. On cardiovascular examination, S1 and S2 were heard, with a pansystolic murmur at the apex and aortic area, along with systolic murmurs over the pulmonary and tricuspid areas. Obstetric examination revealed a distended abdomen with a 32-week-sized gravid uterus and free fluid (Figure 2), while fetal heart sounds were normal. Abdominal ultrasound showed a single fetus in cephalic presentation, with adequate liquor surrounding the fetus, free fluid in the subhepatic region (Figure 3), and an antero-fundally located placenta. Her ECG showed normal sinus rhythm, right axis deviation (RAD), and right ventricular hypertrophy (RVH) (Figure 4).

**Figure 5:**
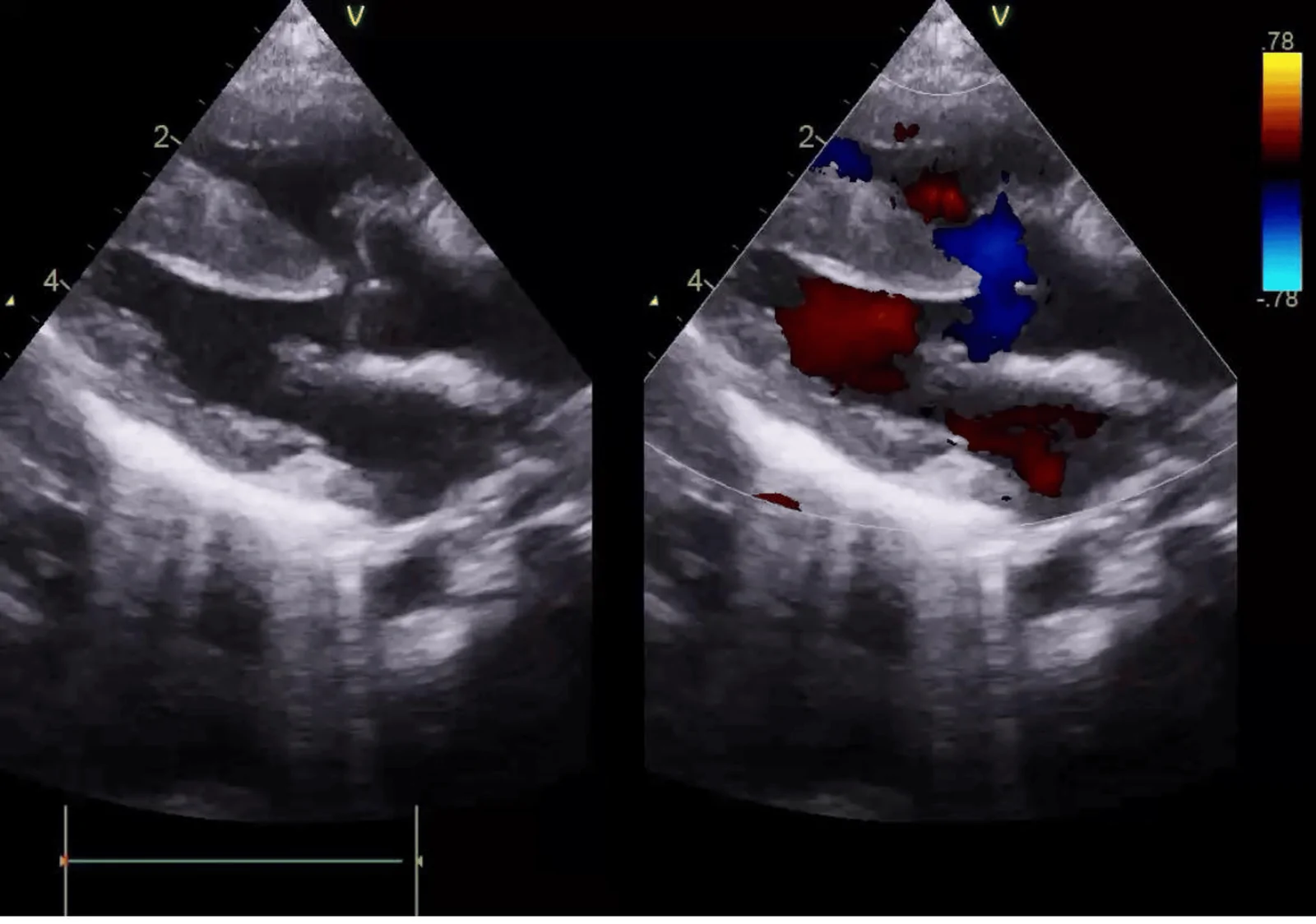
ECHO showing the parasternal long-axis view with a VSD and overriding of the aorta ECHO: echocardiogram; VSD: ventricular septal defect

Urine dipstick testing was performed in the emergency room and showed 4+ proteinuria, leading to a diagnosis of preeclampsia. Laboratory investigations revealed moderate anemia, with a hemoglobin level of 7.8 g/dl. Biochemical evaluation demonstrated deranged liver function tests (LFTs), with elevated serum glutamic-pyruvic transaminase (SGPT) and serum glutamic-oxaloacetic transaminase (SGOT) levels, along with decreased serum albumin and raised serum uric acid levels on kidney function test (KFT) evaluation. Cardiology consultation was obtained, and a screening ECHO was performed, which revealed right ventricular outflow tract (RVOT) obstruction, overriding of the aorta, RVH, and a subaortic VSD, consistent with TOF (Figure 5). However, a right-to-left shunt was also noted, suggesting Eisenmenger physiology.

Pulmonary artery pressure was estimated to be 50-55 mm Hg using Doppler echocardiography. The tricuspid regurgitant jet velocity was measured at 3.4-3.5 m/s, which was also suggestive of Eisenmenger physiology. This elevated pulmonary artery systolic pressure suggested the possibility of additional pulmonary vascular disease rather than RVOT obstruction alone. The patient was admitted and started on tablet sildenafil 20 mg TDS, along with oral antihypertensive therapy (labetalol 100 mg thrice daily), a diuretic, and nifedipine 10 mg once daily. For fetal lung maturity, steroid coverage with an injection of betamethasone was administered.

The patient’s uncontrolled hypertension (150/110 mmHg), proteinuria (4+), edema, deranged liver function, and raised uric acid were consistent with preeclampsia with severe features, necessitating urgent delivery. Epidural analgesia was administered by the anesthesia team, as the patient was initially planned for vaginal delivery. However, due to persistently uncontrolled blood pressure and severe features of preeclampsia, a lower segment cesarean section (LSCS) was performed under general anesthesia, and an alive baby girl weighing 1.560 kg, with an APGAR (Appearance, Pulse, Grimace, Activity, and Respiration) score of 8/10, was delivered. The surgical procedure was completed uneventfully, with perioperative fluid administration carefully monitored through continuous central venous pressure monitoring.

The patient remained vitally stable after surgery and was transferred to the ICU immediately after delivery for observation. Hemodynamic measurements and blood gas analysis were performed on ICU admission, during treatment, and after delivery. Postoperatively, one unit of packed red blood cells (PRBC) was transfused, and the patient was continued on oral sildenafil 20 mg thrice daily in view of PAH. Prophylactic anticoagulation with subcutaneous low-molecular-weight heparin (LMWH) was also initiated. The antihypertensive medication was changed to the calcium channel blocker amlodipine, and her blood pressure was subsequently brought under control. Echocardiography of the baby was normal, with no congenital cardiac abnormality detected.

On postoperative day three, the patient developed fever despite ongoing treatment with ampicillin and amikacin. On examination, the wound was healthy, and the breasts were not engorged. Blood and urine cultures were sent, and empirically, injections of colistin and meropenem were added, along with supplemental oxygen at 3 L/min via nasal cannula. The urine culture was sterile, whereas the blood culture revealed methicillin-susceptible Staphylococcus aureus (MSSA), which was sensitive to clindamycin, linezolid, and vancomycin. Accordingly, the antibiotics were revised, and her condition began to improve following treatment with linezolid. Once she became afebrile, she was shifted to the ward on postoperative day seven. The uterus was well contracted, and she had normal lochia discharge.

Cardiac catheterization was planned; however, the patient’s attendant declined the procedure due to financial constraints and personal reasons. Apart from the postoperative infectious complication of MSSA bacteremia, no major cardiopulmonary decompensation occurred intraoperatively or during the postoperative period. The patient was discharged in stable condition on postoperative day 11, with advice to use barrier contraception strictly and attend the gynaecology and cardiology outpatient departments (OPDs) for follow-up. She was also advised to undergo repeat comprehensive echocardiography after six weeks, along with cardiac MRI. Unfortunately, the patient was subsequently lost to follow-up.

## Discussion

ES is an uncommon congenital cardiac condition seen in about 3% of pregnant patients. [6] The physiological increase in blood volume and reduction in systemic vascular resistance during pregnancy can exacerbate right-to-left shunting, resulting in worsening cyanosis and ventricular dysfunction. Mortality is generally lower in ES associated with ASD or PDA than with VSD, where it may reach 44%. [4,7] Unrepaired TOF may result in Eisenmenger syndrome, as seen in our case. In unrepaired TOF, right-to-left shunting is traditionally attributed to fixed right ventricular outflow tract obstruction rather than increased pulmonary vascular resistance. However, in our patient, Doppler measurement of the tricuspid regurgitant jet velocity, which was 3.4-3.5 m/s, indicated an estimated pulmonary artery systolic pressure of 50-55 mmHg.

This level exceeds what would typically result from RVOT obstruction alone, suggesting the possibility of additional pulmonary vascular disease. [2] Without cardiac catheterization, right-heart hemodynamics and pulmonary vascular resistance could not be directly assessed, and therefore, we could not definitively determine whether the shunt was primarily due to RVOT obstruction or pulmonary vascular remodeling associated with true Eisenmenger syndrome. Consequently, we use the term ‘Eisenmenger physiology’ to describe the overall clinical and hemodynamic condition, namely, a right-to-left shunt with cyanosis and elevated estimated pulmonary artery pressure, rather than to imply the presence of irreversible pulmonary vascular disease. Since cardiac catheterization was not performed, the diagnosis of Eisenmenger physiology in this patient is based on echocardiographic and Doppler findings and should therefore be considered a clinical and echocardiographic diagnosis rather than one confirmed by cardiac catheterization.

The 2025 European Society of Cardiology guidelines strictly advise against pregnancy in women with ES due to the extremely high risks of heart failure, thromboembolic events, and sudden death, which frequently peak during the immediate postpartum period. For affected patients who become pregnant, multidisciplinary management by a Pregnancy Heart Team is mandatory. [8] However, our patient had not received any treatment for her cardiac condition since childhood, and her cardiac condition remained undiagnosed until she consulted a local practitioner two weeks before presentation to our hospital.

Patients who continue their pregnancy or present at an advanced gestational age require intensive antenatal management. Recommended measures include early hospitalization, administration of supplemental oxygen, vasodilators, diuretics, and consideration of prophylactic anticoagulation when appropriate. [4,9] The pregnancy-related physiological decrease in protein S levels, together with an increase in fibrinogen and other procoagulant factors, promotes a hypercoagulable state, which further increases the risk of thrombotic events. Moreover, ES may compound the hypercoagulable state associated with pregnancy, further increasing the risk of microembolic phenomena with significant clinical consequences. Current evidence does not clearly support the routine use of anticoagulation in ES, as it may increase the risk of hemoptysis. Thus, prophylactic anticoagulation with weight-adjusted LMWH or unfractionated heparin (UH) should be considered on an individual basis after careful risk-benefit assessment. [9]

Fetal outcome is closely related to the hematocrit level and maternal arterial oxygen saturation. Favorable pregnancy outcomes are more likely when the hematocrit remains below 65%, and adequate maternal oxygenation is maintained. In women with ES, fetal survival has been reported to reach 92% when oxygen saturation is greater than 90%, whereas it decreases markedly to about 12% when saturation drops below 85%. [9] Loop diuretics should be used cautiously for congestive symptoms, as resulting hypovolemia and hypotension may worsen right ventricular function. Prompt administration of phosphodiesterase type 5 (PDE-5) inhibitors, such as sildenafil, is frequently recommended during pregnancy. In addition, antenatal corticosteroids are commonly administered to women at risk of preterm delivery before 32 weeks of gestation to promote fetal lung maturation and reduce the incidence of respiratory distress syndrome and neonatal mortality. [5,9]

The optimal timing and method of delivery in patients with ES and PAH remain controversial. Vaginal delivery is the preferred mode of delivery if there is no obstetric indication for cesarean section. During vaginal delivery in such patients, efforts should be directed toward maintaining adequate oxygen saturation, providing effective analgesia, avoiding hypotension, administering intrapartum endocarditis prophylaxis, and minimizing the second stage of labor through assisted delivery when appropriate. Moreover, cesarean delivery is associated with a substantially higher risk of maternal morbidity and mortality than vaginal birth, with reported rates of 75% and 34%, respectively. [4,7,9] Existing literature suggests that cesarean delivery in ES does not confer maternal benefit, whereas vaginal delivery is associated with less blood loss and a lower risk of infection.

However, in selected patients, including those with severe PAH and significant obstetric complications, cesarean delivery may facilitate better control of maternal cardiovascular stress. It can prevent the sudden cardiovascular shifts associated with labor, which may elevate pulmonary vascular resistance and cause fatal decompensation. Surgical management also facilitates the presence of senior multidisciplinary staff and allows for controlled hemodynamics, targeted pulmonary vasodilator administration, and meticulous timing of anticoagulation to balance the severe risks of thrombosis and postpartum hemorrhage. [9] In our patient, the presence of ES with high PAH and preeclampsia with severe features led to the decision to proceed with cesarean delivery. Recent advances in pharmacological therapy, multidisciplinary management, and neonatal care have significantly improved survival among pregnant women with ES. As most pregnancy-related cardiovascular changes stabilize within two weeks postpartum, intensive postoperative care is essential, particularly during the first postpartum week, when the risk of complications is greatest. [9,10]

Family members and primary care physicians should be educated to recognize early symptoms of congenital heart disease, such as exercise intolerance, dyspnea, and cyanosis, particularly when these symptoms are present from childhood. Primary care clinicians should maintain a high index of suspicion, perform a basic evaluation, including clinical examination, oxygen saturation measurement, and CBC, and refer patients for echocardiography when indicated. Preconception counseling and contraception should be emphasized, as ES is an absolute contraindication to pregnancy. Women with PAH/ES should receive clear, documented counseling regarding the high maternal and fetal risks and should be offered effective contraception. If pregnancy occurs or is continued despite counseling, primary care physicians should recognize that early involvement of a multidisciplinary team, including cardiology with pulmonary hypertension expertise, obstetrics, anesthesia, neonatology, and hematology, is essential. Primary care follow-up should ensure coordination of specialist cardiology care, optimization of PAH therapy, infection management, correction of anemia, contraception counseling, and psychosocial support.

In a study, Gangakhedkar et al. reported successful management of a case of ES secondary to malaligned inlet VSD with bidirectional shunt and severe PS in a 32-year-old third gravida woman with hearing and speech impairment, which posed a barrier to effective communication between the patient and the treating physicians. She underwent cesarean section at 35 weeks of gestation and delivered a healthy male child with no congenital anomalies. [10] Benlamkaddem et al., in their study, reported a case of ES secondary to PDA with severe preeclampsia in a 23-year-old primigravida at the 34th week of gestation, resulting in fatal maternal and fetal outcomes. [11] While early booking and referral to a tertiary-level centre, with regular antenatal visits, are recommended in such cases of ES, our patient had neither undergone early antenatal booking nor attended regular antenatal visits and remained undiagnosed with cardiac disease until an advanced gestational age in the current pregnancy. Fortunately, she was appropriately managed promptly, and no complications occurred during or after the cesarean section, resulting in a favorable maternal and fetal outcome.

## Conclusions

ES is a rare yet potentially life-threatening condition during pregnancy, associated with significant maternal and fetal morbidity and mortality. While early pregnancy termination is recommended, some women may choose to conceive and continue the pregnancy despite being aware of the associated risks. This case report illustrates that, despite the high risk, favorable maternal and neonatal outcomes may be achieved through prompt recognition, individualized management, and a coordinated multidisciplinary approach. For primary care providers, the priorities are early detection of congenital heart disease, effective preconception counseling, provision of safe and effective contraception, and prompt referral when pregnancy occurs, before symptoms worsen.

## References

[1] Yuan SM (2016). Eisenmenger syndrome in pregnancy. Braz J Cardiovasc Surg.

[2] Basit H, Wallen TJ, Sergent BN (2026). Eisenmenger Syndrome. StatPearls [Internet]. Treasure Island (FL): Jan.

[3] Kumar N, Kumar A, Sneha K, Agarwal M, Kumar S (2019). Eisenmenger syndrome and emergency laparotomy: a case report. Egypt J Cardiothorac Anesth.

[4] Duan R, Xu X, Wang X (2016). Pregnancy outcome in women with Eisenmenger's syndrome: a case series from west China. BMC Pregnancy Childbirth.

[5] Liu Y, Li Y, Zhang J (2023). Pregnancy outcomes of women with Eisenmenger syndrome: a single-center study. Int J Cardiol.

[6] Rathod S, Samal SK (2014). Successful pregnancy outcome in a case of eisenmenger syndrome: a rare case report. J Clin Diagn Res.

[7] Alsomali F, Mushtaq S, Bakir M, Almustanyir S (2022). Fruitful pregnancy outcome in a case of Eisenmenger syndrome with severe pulmonary hypertension: a rare case report. Cureus.

[8] Arbustini E, Beneventi F, Bozzani A (2026). ESC guidelines 2025 on cardiovascular disease and pregnancy: considerations and future perspectives. Eur Heart J Suppl.

[9] Slaibi A, Ibraheem B, Mohanna F (2021). Challenging management of a pregnancy complicated by Eisenmenger syndrome; a case report. Ann Med Surg (Lond).

[10] Gangakhedkar GR, Chhabria RD, Gour SD, Palani Y (2020). Emergency caesarean section of a patient with Eisenmenger's syndrome: a tight-rope walk. Ann Card Anaesth.

[11] Benlamkaddem S, Bouyermane F, Doughmi D, Berdai MA, Harandou M (2023). Fatal association of Eisenmenger syndrome and severe preeclampsia. Cureus.

